# Supplementary material for: HIVprotI: an integrated web based platform for prediction and design of HIV proteins inhibitors
Source: J Cheminform. 2018 Mar 9;10:12. doi: 10.1186/s13321-018-0266-y (PMC5845081; doi:10.1186/s13321-018-0266-y)
Supplement: Supplementary file 2 — Additional file 2. Source code of HIVProtI web server. [file 13321_2018_266_MOESM2_ESM.zip › HIVprotI_Source-code/hivprotI/weka-3-6-6/doc/weka/filters/unsupervised/attribute/AbstractTimeSeries.html]

AbstractTimeSeries


---


|  |  |  |
| --- | --- | --- |
| **Overview** \| **Package** \| **Class** \| **Tree** \| **Deprecated** \| **Index** \| **Help** \| **Weka's home** | |  |
| PREV CLASS   **NEXT CLASS** | **FRAMES**    **NO FRAMES**     **All Classes** |  |
| SUMMARY: NESTED \| FIELD \| CONSTR \| METHOD | DETAIL: FIELD \| CONSTR \| METHOD |  |


---


## weka.filters.unsupervised.attribute Class AbstractTimeSeries

```
java.lang.Object
  weka.filters.Filter
      weka.filters.unsupervised.attribute.AbstractTimeSeries
```

**All Implemented Interfaces:**: java.io.Serializable, CapabilitiesHandler, OptionHandler, RevisionHandler, UnsupervisedFilter

**Direct Known Subclasses:**: TimeSeriesTranslate

---

``` public abstract class AbstractTimeSeries extends Filter implements UnsupervisedFilter, OptionHandler ```

An abstract instance filter that assumes instances form time-series data and
performs some merging of attribute values in the current instance with
attribute attribute values of some previous (or future) instance. For
instances where the desired value is unknown either the instance may
be dropped, or missing values used.

Valid filter-specific options are:

-R index1,index2-index4,...  
Specify list of columns to calculate new values for.
First and last are valid indexes.
(default none)

-V   
Invert matching sense (i.e. calculate for all non-specified columns)

-I num   
The number of instances forward to merge values between.
A negative number indicates taking values from a past instance.
(default -1)

-M   
For instances at the beginning or end of the dataset where the translated
values are not known, remove those instances (default is to use missing
values).

**Version:**
:   $Revision: 1.8 $

**Author:**
:   Len Trigg (trigg@cs.waikato.ac.nz)

**See Also:**: Serialized Form

---

**Constructor Summary**

`AbstractTimeSeries()`


| **Method Summary** | |
| --- | --- |
| `java.lang.String` | `attributeIndicesTipText()`             Returns the tip text for this property |
| `boolean` | `batchFinished()`             Signifies that this batch of input to the filter is finished. |
| `java.lang.String` | `fillWithMissingTipText()`             Returns the tip text for this property |
| `java.lang.String` | `getAttributeIndices()`             Get the current range selection |
| `boolean` | `getFillWithMissing()`             Gets whether missing values should be used rather than removing instances where the translated value is not known (due to border effects). |
| `int` | `getInstanceRange()`             Gets the number of instances forward to translate values between. |
| `boolean` | `getInvertSelection()`             Get whether the supplied columns are to be removed or kept |
| `java.lang.String[]` | `getOptions()`             Gets the current settings of the filter. |
| `boolean` | `input(Instance instance)`             Input an instance for filtering. |
| `java.lang.String` | `instanceRangeTipText()`             Returns the tip text for this property |
| `java.lang.String` | `invertSelectionTipText()`             Returns the tip text for this property |
| `java.util.Enumeration` | `listOptions()`             Returns an enumeration describing the available options. |
| `void` | `setAttributeIndices(java.lang.String rangeList)`             Set which attributes are to be copied (or kept if invert is true) |
| `void` | `setAttributeIndicesArray(int[] attributes)`             Set which attributes are to be copied (or kept if invert is true) |
| `void` | `setFillWithMissing(boolean newFillWithMissing)`             Sets whether missing values should be used rather than removing instances where the translated value is not known (due to border effects). |
| `boolean` | `setInputFormat(Instances instanceInfo)`             Sets the format of the input instances. |
| `void` | `setInstanceRange(int newInstanceRange)`             Sets the number of instances forward to translate values between. |
| `void` | `setInvertSelection(boolean invert)`             Set whether selected columns should be removed or kept. |
| `void` | `setOptions(java.lang.String[] options)`             Parses a given list of options controlling the behaviour of this object. |

**Methods inherited from class weka.filters.Filter**

`batchFilterFile, filterFile, getCapabilities, getCapabilities, getOutputFormat, getRevision, isFirstBatchDone, isNewBatch, isOutputFormatDefined, main, makeCopies, makeCopy, numPendingOutput, output, outputPeek, toString, useFilter, wekaStaticWrapper`

**Methods inherited from class java.lang.Object**

`equals, getClass, hashCode, notify, notifyAll, wait, wait, wait`

**Constructor Detail**

### AbstractTimeSeries

```
public AbstractTimeSeries()
```


**Method Detail**

### listOptions

```
public java.util.Enumeration listOptions()
```

:   Returns an enumeration describing the available options.

    :   **Specified by:**: `listOptions` in interface `OptionHandler`
    :   **Returns:**: an enumeration of all the available options.

---


### setOptions

```
public void setOptions(java.lang.String[] options)
                throws java.lang.Exception
```

:   Parses a given list of options controlling the behaviour of this object.
    Valid options are:

    -R index1,index2-index4,...  
    Specify list of columns to copy. First and last are valid indexes.
    (default none)

    -V  
    Invert matching sense (i.e. calculate for all non-specified columns)

    -I num   
    The number of instances forward to translate values between.
    A negative number indicates taking values from a past instance.
    (default -1)

    -M   
    For instances at the beginning or end of the dataset where the translated
    values are not known, remove those instances (default is to use missing
    values).

    :   **Specified by:**: `setOptions` in interface `OptionHandler`
    :   **Parameters:**: `options` - the list of options as an array of strings **Throws:**: `java.lang.Exception` - if an option is not supported

---


### getOptions

```
public java.lang.String[] getOptions()
```

:   Gets the current settings of the filter.

    :   **Specified by:**: `getOptions` in interface `OptionHandler`
    :   **Returns:**: an array of strings suitable for passing to setOptions

---


### setInputFormat

```
public boolean setInputFormat(Instances instanceInfo)
                       throws java.lang.Exception
```

:   Sets the format of the input instances.

    :   **Overrides:**: `setInputFormat` in class `Filter`
    :   **Parameters:**: `instanceInfo` - an Instances object containing the input instance structure (any instances contained in the object are ignored - only the structure is required). **Returns:**: true if the outputFormat may be collected immediately **Throws:**: `java.lang.Exception` - if the format couldn't be set successfully

---


### input

```
public boolean input(Instance instance)
              throws java.lang.Exception
```

:   Input an instance for filtering. Ordinarily the instance is processed
    and made available for output immediately. Some filters require all
    instances be read before producing output.

    :   **Overrides:**: `input` in class `Filter`
    :   **Parameters:**: `instance` - the input instance **Returns:**: true if the filtered instance may now be collected with output(). **Throws:**: `java.lang.Exception` - if the input instance was not of the correct format or if there was a problem with the filtering.

---


### batchFinished

```
public boolean batchFinished()
```

:   Signifies that this batch of input to the filter is finished. If the
    filter requires all instances prior to filtering, output() may now
    be called to retrieve the filtered instances.

    :   **Overrides:**: `batchFinished` in class `Filter`
    :   **Returns:**: true if there are instances pending output **Throws:**: `java.lang.IllegalStateException` - if no input structure has been defined

---


### fillWithMissingTipText

```
public java.lang.String fillWithMissingTipText()
```

:   Returns the tip text for this property

    :   **Returns:**: tip text for this property suitable for displaying in the explorer/experimenter gui

---


### getFillWithMissing

```
public boolean getFillWithMissing()
```

:   Gets whether missing values should be used rather than removing instances
    where the translated value is not known (due to border effects).

    :   **Returns:**: true if so

---


### setFillWithMissing

```
public void setFillWithMissing(boolean newFillWithMissing)
```

:   Sets whether missing values should be used rather than removing instances
    where the translated value is not known (due to border effects).

    :   **Parameters:**: `newFillWithMissing` - true if so

---


### instanceRangeTipText

```
public java.lang.String instanceRangeTipText()
```

:   Returns the tip text for this property

    :   **Returns:**: tip text for this property suitable for displaying in the explorer/experimenter gui

---


### getInstanceRange

```
public int getInstanceRange()
```

:   Gets the number of instances forward to translate values between.
    A negative number indicates taking values from a past instance.

    :   **Returns:**: Value of InstanceRange.

---


### setInstanceRange

```
public void setInstanceRange(int newInstanceRange)
```

:   Sets the number of instances forward to translate values between.
    A negative number indicates taking values from a past instance.

    :   **Parameters:**: `newInstanceRange` - Value to assign to InstanceRange.

---


### invertSelectionTipText

```
public java.lang.String invertSelectionTipText()
```

:   Returns the tip text for this property

    :   **Returns:**: tip text for this property suitable for displaying in the explorer/experimenter gui

---


### getInvertSelection

```
public boolean getInvertSelection()
```

:   Get whether the supplied columns are to be removed or kept

    :   **Returns:**: true if the supplied columns will be kept

---


### setInvertSelection

```
public void setInvertSelection(boolean invert)
```

:   Set whether selected columns should be removed or kept. If true the
    selected columns are kept and unselected columns are copied. If false
    selected columns are copied and unselected columns are kept.

    :   **Parameters:**: `invert` - the new invert setting

---


### attributeIndicesTipText

```
public java.lang.String attributeIndicesTipText()
```

:   Returns the tip text for this property

    :   **Returns:**: tip text for this property suitable for displaying in the explorer/experimenter gui

---


### getAttributeIndices

```
public java.lang.String getAttributeIndices()
```

:   Get the current range selection

    :   **Returns:**: a string containing a comma separated list of ranges

---


### setAttributeIndices

```
public void setAttributeIndices(java.lang.String rangeList)
```

:   Set which attributes are to be copied (or kept if invert is true)

    :   **Parameters:**: `rangeList` - a string representing the list of attributes. Since the string will typically come from a user, attributes are indexed from 1. eg: first-3,5,6-last

---


### setAttributeIndicesArray

```
public void setAttributeIndicesArray(int[] attributes)
```

:   Set which attributes are to be copied (or kept if invert is true)

    :   **Parameters:**: `attributes` - an array containing indexes of attributes to select. Since the array will typically come from a program, attributes are indexed from 0.


---


|  |  |  |
| --- | --- | --- |
| **Overview** \| **Package** \| **Class** \| **Tree** \| **Deprecated** \| **Index** \| **Help** \| **Weka's home** | |  |
| PREV CLASS   **NEXT CLASS** | **FRAMES**    **NO FRAMES**     **All Classes** |  |
| SUMMARY: NESTED \| FIELD \| CONSTR \| METHOD | DETAIL: FIELD \| CONSTR \| METHOD |  |


---
